# Expression of Neutral Endopeptidase, Endothelin-1, and Nuclear Factor Kappa B in Prostate Cancer: Interrelations and Associations with Prostate-Specific Antigen Recurrence after Radical Prostatectomy

**DOI:** 10.1155/2012/452795

**Published:** 2012-05-15

**Authors:** Panagiotis J. Vlachostergios, Foteini Karasavvidou, Grigorios Kakkas, George Moutzouris, Anna Patrikidou, Ioannis A. Voutsadakis, Danai D. Daliani, Elias Zintzaras, Michael D. Melekos, Christos N. Papandreou

**Affiliations:** ^1^Department of Medical Oncology, University Hospital of Larissa, University of Thessaly School of Medicine, 41110 Larissa, Greece; ^2^Department of Pathology, University Hospital of Larissa, University of Thessaly School of Medicine, 41110 Larissa, Greece; ^3^Department of Urology, University Hospital of Larissa, University of Thessaly School of Medicine, 41110 Larissa, Greece; ^4^Department of Medicine, Institut Gustave Roussy, 94805 Villejuif, France; ^5^Centre Pluridisciplinaire d'Oncologie, Centre Hospitalier Universitaire Vaudois, 1101Lausanne, Switzerland; ^6^Department of Biomathematics, University of Thessaly School of Medicine, 41222 Larissa, Greece; ^7^Institute for Clinical Research and Health Policy Studies, Tufts Medical Center and Tufts University School of Medicine, Boston, MA 02111, USA

## Abstract

*Objective*. To study the impact of the neutral endopeptidase (NEP)/neuropeptides (NPs) axis and nuclear factor kappa B (NF*κ*B) as predictors of prostate-specific antigen (PSA) recurrence after radical prostatectomy (RP). *Patients and Methods*. 70 patients with early-stage PC were treated with RP and their tumor samples were evaluated for expression of NEP, endothelin-1 (ET-1) and NF*κ*B (p65). Time to PSA recurrence was correlated with the examined parameters and combined with preoperative PSA level, Gleason score, pathological TNM (pT) stage, and surgical margin (SM) assessment. *Results and Limitations*. Membranous expression of NEP (*P* < 0.001), cytoplasmic ET-1 (*P* = 0.002), and cytoplasmic NF*κ*B (*P* < 0.001) were correlated with time to PSA relapse. NEP was associated with ET-1 (*P* < 0.001) and NF*κ*B (*P* < 0.001). ET-1 was also correlated with NF*κ*B (*P* < 0.001). NEP expression (*P* = 0.017), pT stage (*P* = 0.013), and SMs (*P* = 0.036) were independent predictors of time to PSA recurrence. *Conclusions*. There seems to be a clinical model of NEP/NPs and NF*κ*B pathways interconnection, with their constituents following inverse patterns of expression in accordance with their biological roles and molecular interrelations.

## 1. Introduction

Neuropeptides (NPs) constitute a family of potent vasoconstrictor peptides with mitogenic properties relevant to carcinogenesis. Endothelin-1 (ET-1) is a major representative, consistently implicated in prostate cancer (PC) progression through induction of proliferation of PC cells *in vitro*, while i*n vivo*, increased ET-1 levels were detected in plasma and tissue specimens from patients with castrate-resistant PC [1–3]. 

The enzyme responsible for cleavage and inactivation of ET-1 and other bioactive NPs is neutral endopeptidase (NEP or CD10). NEP is a cell surface peptidase normally expressed by various tissues, including prostate [4, 5] but its loss of expression has been correlated with tumor progression to castration resistance by allowing NPs growth-promoting effects [6].

Nuclear factor kappa B (NF*κ*B) is a transcription factor known for its prosurvival and antiapoptotic roles in various types of neoplasia. The most studied form of NF*κ*B is the heterodimer formed by the p50 and RelA (p65) proteins. Emerging preclinical evidence implicates NF*κ*B in growth, survival, angiogenesis, and metastatic progression of PC cells [7, 8]. Constitutive activation of NF*κ*B has been detected in castrate-resistant PC xenografts and in PC tissues [9].

Since the continuously increasing list of prognostic biomarkers candidate for clinical use in PC and given the central role played by the NEP/NPs and NF*κ*B pathways in PC progression, gross data from immunohistochemical studies of radical prostatectomy (RP) specimens has accumulated. However, the existence of conflicting results as well as the lack of an integrated investigation of both pathways in the same study still hampers proper interpretation of data towards refining the already existing prognostic models of the disease.

## 2. Patients and Methods

### 2.1. Patient Selection

The study enrolled patients over 18 years old with histologically newly diagnosed, early-stage PC, admitted to the Department of Urology, University Hospital of Larissa. All patients of the study underwent an open retropubic radical prostatectomy. Patients were hormone and treatment (chemotherapy and radiotherapy) naïve at the time of surgery. No history of previous reproductive or endocrine diseases was reported. The study was approved by the Internal Review Board of our institution and written informed consent was provided by all patients before study entry.

Patient demographics (age) as well as clinico-pathological parameters, including preoperative PSA level, pT stage and Gleason score of the primary tumor, lymph node status, SMs, PSA recurrence, and survival data were recorded.

Hematoxylin and eosin-stained tissue sections from 70 RP specimens were examined by a single, blinded histopathologist, based on the availability of both adequate followup and representative pathology specimens. The radical prostatectomy specimens were processed using a whole-mount technique. Evaluation of histopathological characteristics was made according to recommendations of the 2004 World-Health-Organization-(WHO-) sponsored International Consultation on Prediction of Patients Outcome in Prostate Cancer meeting [10]. Cases were divided into 2 Gleason groups: low (≤3 + 4; *n* = 50) and high (≥4 + 3; *n* = 20), as there were no lower Gleason score (2, 3, 4) samples based on the established 3-group histopathological criteria of current literature (low, medium, and high). Cases were also grouped according to pT stage into either organ-confined disease (pT ≤ 2; *n* = 42) or advanced tumors extending beyond the prostatic capsule (pT ≥ 3; *n* = 28). pT3 group consisted of 11 patients with pT3a disease and 16 patients of pT3b stage; however due to the small number of patients, no additional subgroup analysis was performed. With regard to preoperative PSA levels, patients were categorized in 2 subgroups: <10 ng/mL and ≥10 ng/mL. The majority of patients had a preoperative PSA level <10 ng/mL (*n* = 60), while they also displayed negative SM (*n* = 49) and lymph node status (*n* = 53). The latter was not included in most of our statistical analyses due to missing information regarding a significant number of patients (*n* = 11 or 15.7%). Patients' clinical and pathological characteristics are depicted in Table 1.

### 2.2. Immunohistochemical (IHC) Procedures

The RP specimens were fixed in 10% buffered formalin solution and embedded in paraffin blocks. Serial sections (4 **μ*m*) from selected 1 or 2 paraffin blocks of each case were obtained. Tissue blocks were chosen based on the presence of both, the primary and the secondary architectural Gleason pattern of prostate adenocarcinoma, as determined on hematoxylin and eosin sections. Sections were deparaffinised in xylene and rehydrated through decreasing alcohols. Antigen unmasking for NF*κ*B was achieved by treating sections in a 6 mM citrate buffer (pH 6) for a total of 20 min in a microwave oven at 850 Watt. Antigen unmasking for NEP and ET-1 was achieved by boiling sections in Trilogy reagent (Cell Marque, Rocklin, CA) for a total of 1 hour in a commercially available steamer. After quenching endogenous peroxidase with 3% hydrogen peroxide solution for 10 min, slides were incubated at room temperature for 30 minutes with the following primary mouse monoclonal antibodies: against p65 subunit of NF*κ*B (clone F-6, 1 : 500 dilution, Santa Cruz Biotechnology, Inc., CA) and anti-NEP (clone 56C6, 1 : 30 dilution, DAKO, Denmark). Adjacent sections were incubated overnight at 4°C with mouse monoclonal antibody against ET-1 (clone TRET-485, 1 : 100 dilution, SIGMA, UK). Staining was developed with substrate chromogen solution (EnVision, DAKO, Glostrup, Denmark) and diaminobenzidine for 10 minutes. Slides were counterstained with Harris hematoxylin for 1 minute, dehydrated, and mounted with DPX solution.

### 2.3. Assessment of IHC Staining

Assessment of IHC staining was made according to evaluations in previous studies. NF*κ*B immunostaining was cytoplasmic. NEP immunostaining was membranous and apical cytoplasmic. ET-1 immunostaining was cytoplasmic. Intensity of immunostaining for all 3 examined parameters was evaluated, using a score from 0 to 3 (0: no staining 1: weak staining, 2: moderate staining, and 3: strong staining) compared with the background [9, 11, 12]. Weak and moderate staining (0–2) versus strong staining (3) were considered for statistical analysis of NF*κ*B and ET-1, classified as low and high expression, respectively [9, 12]. High NEP expression was defined as a score of 2 or greater (2-3) and low expression as a score of less than 2 (0-1) [11].

The weak ET-1 staining category was assessed to be less than that of endothelial cells, the moderate ET-1 staining category was determined to be equal to that of endothelial cells, and the intense ET-1 staining category exhibited more than that of endothelial cells. The normal adjacent prostate gland was used as an internal control marker for the evaluation of NEP and NF*κ*B expression. IHC reaction was glandular for all tested parameters (NEP, ET-1, and NF*κ*B).

### 2.4. Study Endpoints

Our objective was to investigate possible interrelations between IHC expression of NEP, ET-1, and NF*κ*B as well as their potential correlations with preoperative PSA level, Gleason score, pT stage, and SMs in patients with hormone naïve PC undergoing RP. We further examined the putative prognostic role of these parameters in association with time to PSA failure. The response variable, time to PSA recurrence, was defined as the time from RP to the time of the first detectable (nonzero) PSA measurement >0.2 ng/mL. 

### 2.5. Statistical Analysis

The Fisher's and *χ* ^2^ tests were used to explore associations between NEP, ET-1, NF*κ*B expression and Gleason score, tumor stage, and SM status. Pearson's correlation coefficient was reported to determine the correlation between NEP, ET-1, NF*κ*B expression and preoperative PSA levels. The Kaplan-Meier method was used to determine the effect of each categorical variable on PSA relapse-free survival, and the log-rank test was used to compare PSA relapse-free survival differences within each variable. For PSA recurrence-free survival analysis at the univariate and multivariate level, the Cox proportional hazards model was used to estimate hazard ratios (HR) with 95% confidence intervals (CI). Statistical significance was determined by using two-tailed *P*  values and was reported at *P* < 0.05 level. Statistical analysis was performed using SPSS (SPSS for Windows, version 15.0, SPSS, Chicago, IL).

## 3. Results

Thirty-eight (54%) patients developed PSA recurrence during followup and 32 (46%) did not have a PSA relapse. Two patients (2.8%) expired. The estimated median follow-up time, as calculated by the reverse Kaplan-Meier method was 30 months (range 12–86) while the median time to PSA recurrence was 56 months (range 1–74). Among relapsed patients, 22 received combined androgen blockade (CAB), 1 received local radiotherapy, and 9 patients were treated with a combination of CAB and radiotherapy (5 in sequential order and 4 concurrently).

### 3.1. NEP Is Associated with Grade, Stage, and Time to PSA Relapse

According to level of NEP expression, patients were divided into a group of low (*n* = 36, 51.4%) and another of high NEP IHC expression (*n* = 34, 48.6%). In univariate analysis, we observed a significant association of NEP with Gleason score, as the majority of high NEP-expressing tumors (30/34 or 88.2%) correlated with low Gleason score (*P* = 0.003). Similar was the case for the association between NEP and pT stage, with 25/34 or approximately 73.5% of tumors with high NEP expression correlating with low pT stage (*P* = 0.030) (Table 2).

**Figure 1:**
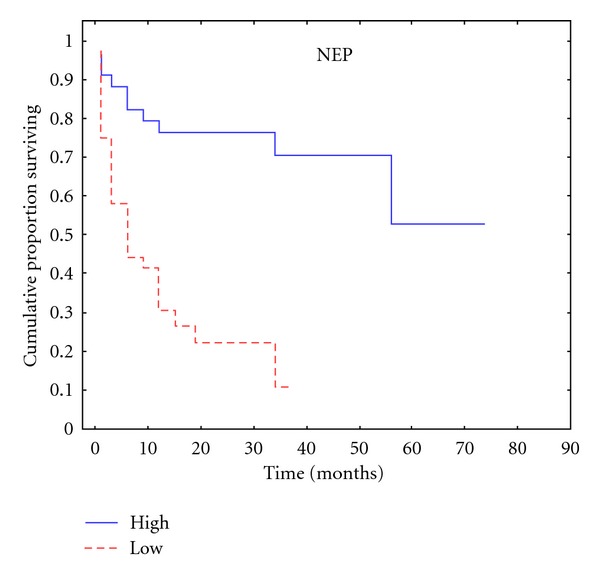
Kaplan-Meier plot according to NEP expression for time to PSA relapse.

The expression of NEP was found to be associated with time to PSA recurrence (*r* = 0.485), (*P* < 0.001) (Table 2, Figure 1). There were no statistically significant correlations between NEP and preoperative PSA levels (*P* = 0.076) or NEP and SMs (*P* = 0.121) (Table 2).

### 3.2. ET-1 Is Associated with Grade, Stage, and Time to PSA Relapse

**Figure 2:**
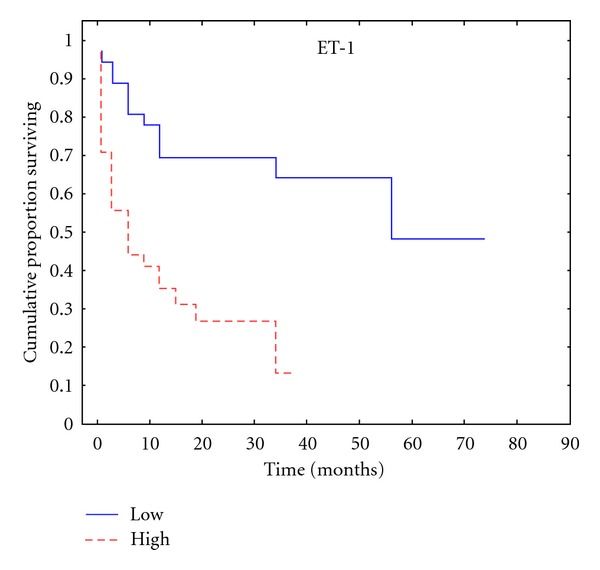
Kaplan-Meier plot according to ET-1 expression for time to PSA relapse.

ET-1 expression was equally divided in two groups of either low (*n* = 34, 48.6%) or high (*n* = 36, 51.4%) immunoreactivity. Elevated ET-1 expression was correlated with more advanced disease, evidenced by both pT stage (*P* = 0.003) and Gleason score (*P* = 0.008) in univariate analysis (Table 2). ET-1 was also found to be an indicator of biochemical progression as its expression correlated with a smaller time interval from RP until PSA relapse (*r* = −0.375), (*P* < 0.001) (Table 2, Figure 2). There were no statistically significant correlations between ET-1 and preoperative PSA levels (*P* = 0.277) or ET-1 and SMs (*P* = 0.068) (Table 2).

### 3.3. NF*κ*B Expression Is Correlated with Grade, Stage, and Time to PSA Relapse

**Figure 3:**
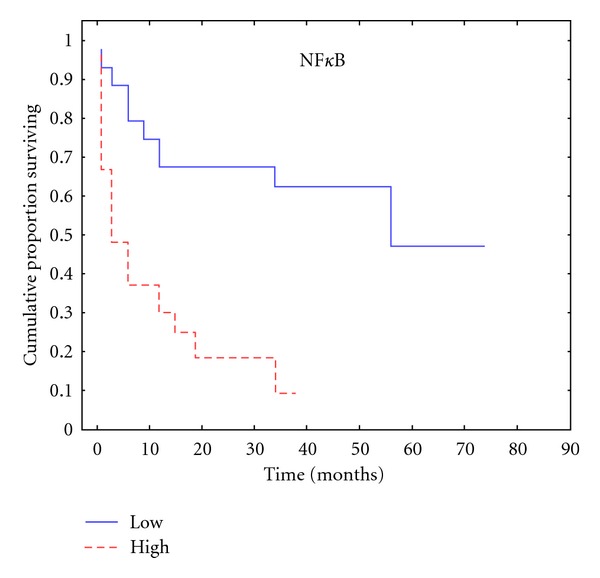
Kaplan-Meier plot according to NF*κ*B expression for time to PSA relapse.

NF*κ*B staining was found to be low in 60% of patients (*n* = 42) whereas high in 40% of the cohort (*n* = 28). There was an association of NF*κ*B expression with both stage (*P* = 0.003) and grade (*P* = 0.002) in univariate analysis (Table 2). High NF*κ*B expression was also found to significantly correlate with a shortened time to PSA recurrence (*r* = −0.432), (*P* < 0.001) (Table 2, Figure 3). There were no statistically significant correlations between NF*κ*B and preoperative PSA levels (*P* = 0.191) or NF*κ*B and SMs (*P* = 0.180) (Table 2).

### 3.4. NEP Expression Is Associated with ET-1 and NF*κ*B Expression

**Figure 4:**
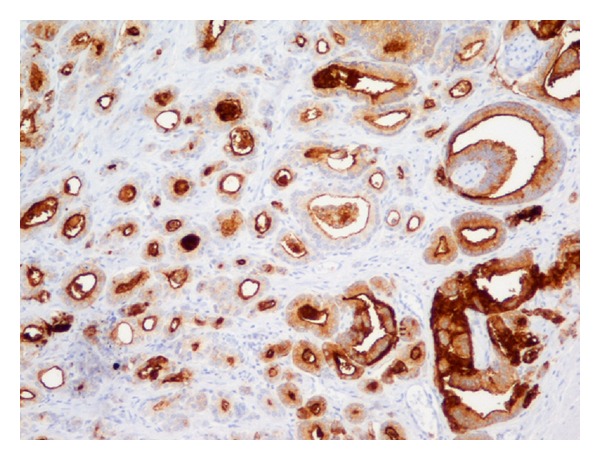
Prostate adenocarcinoma Gleason pattern 3. Strong membranous and apical cytoplasmic IHC staining for NEP (×200).

**Figure 5:**
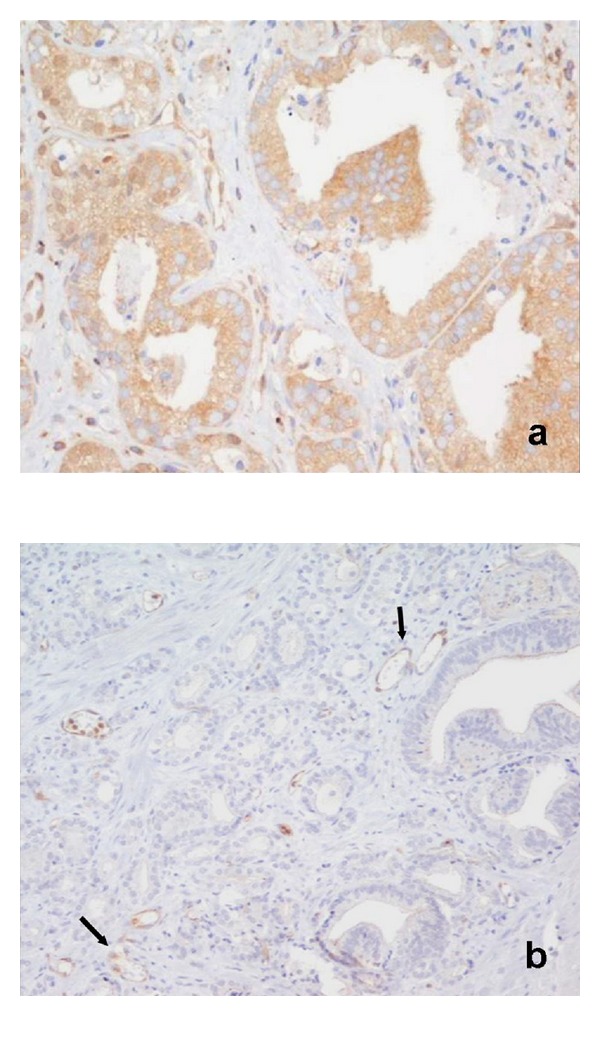
Prostate adenocarcinoma Gleason pattern 3. The same area as in Figure 4. (a) Weak-to-moderate cytoplasmic IHC staining for NF*κ*B (×400); (b) Negative immunoreactivity for ET-1. Positive marker the capillary endothelium (arrows) (×200).

**Figure 6:**
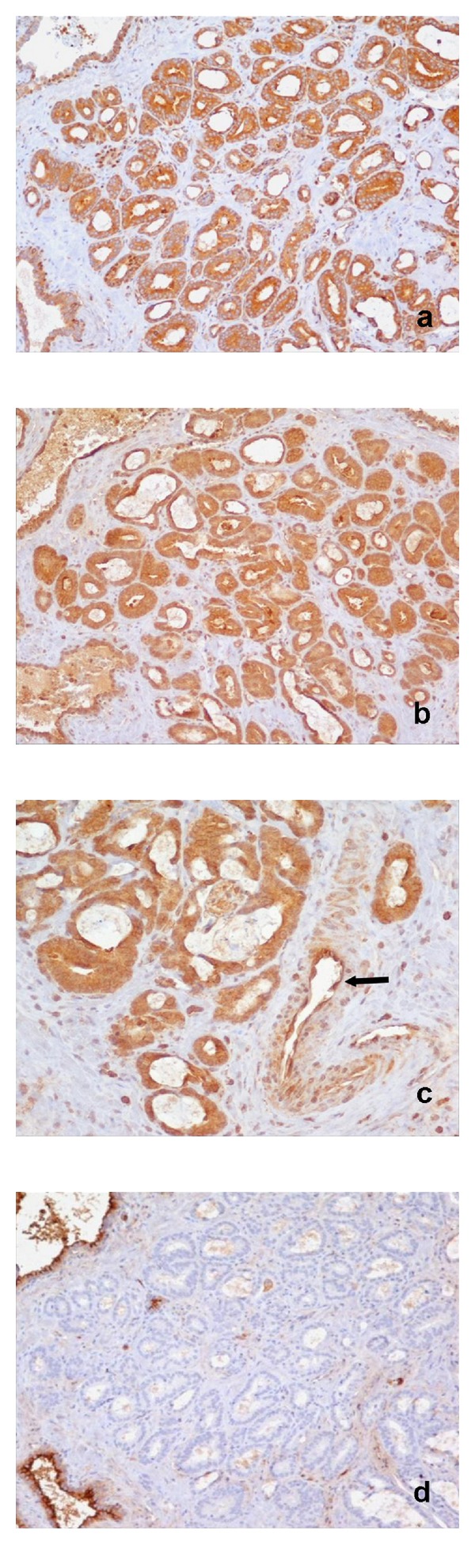
Prostate adenocarcinoma Gleason pattern 3. (a) Strong cytoplasmic IHC staining for NF*κ*B (×200); (b) strong cytoplasmic IHC staining for ET-1 (×200); (c) strong cytoplasmic IHC staining for ET-1. Positive marker the capillary endothelium (arrow) (×400); (d) negative immunoreactivity for NEP. Positive marker the normal prostate glands (×200).

In univariate analysis, a significant correlation was found between IHC expression of NEP and ET-1 (*P* < 0.001). Same was the case for the NEP-NF*κ*B relationship (*P* < 0.001) (Table 3). ET-1 and NF*κ*B expressions were also interrelated (*P* < 0.001). IHC expression patterns of NEP, ET-1, and NF*κ*B are depicted in Figures 4, 5 and 6. 

### 3.5. Other Univariate Correlations

pT stage (*P* < 0.001), Gleason score (*P* < 0.001), and SM status (*P* = 0.004) were related with PSA relapse-free survival (Table 1). There was no statistically significant correlation between age (*P* = 0.277), preoperative PSA (*P* = 0.143) or lymph node status (*P* = 0.072), and time to PSA recurrence, respectively (Table 1). Preoperative PSA level was associated with pT stage (*P* = 0.016) but not with Gleason score (*P* = 0.123). Stage and grade were interrelated (*P* < 0.001).

### 3.6. NEP, pT Stage and SMs Are Independent Predictors of Time to PSA Relapse

In multivariate analysis, there was a significant association between NEP expression and time to PSA recurrence after controlling for preoperative PSA, tumor stage, Gleason score, SMs, NF*κ*B, and ET-1 (*P* = 0.017; 95% [CI] = 0.228 [0.068–0.766]). pT stage (*P* = 0.013; 95% [CI] = 3.025 [1.257–7.277] and SM status (*P* = 0.036; 95% [CI] = 2.061 [1.049–4.051]) also retained their significance as predictors of time to PSA recurrence (Table 4).

## 4. Discussion

In this study we have simultaneously examined the expression of major components of two systems that have separately been correlated with PC progression, the NEP/NPs, and the NF*κ*B pathway.

We have confirmed the previously reported association between expression of components of the endothelin axis and stage, grade [11, 13, 14]. We have also observed an association of ET-1 expression with time to PSA recurrence, in accordance with results from Rosenblatt et al. [12] who demonstrated that both the intensity and the combination product of intensity and extent of ET-1 immunoreactivity (IRp) but not the staining extent alone predicted biochemical relapse in a large-scale study of 287 PC specimens from RP. Recurrence-free survival in patients with strong ET-1 staining was shorter than in those with weaker expression [12].

Moreover, we have observed that the aggressiveness of the examined tumors as evidenced by increasing grade and advanced stage coincided with loss of membranous NEP, justifying the latter's biological role in attenuation of oncogenic signaling induced by NEP substrates. Accordingly, a significant association was observed between NEP and time to PSA recurrence.

The prognostic relevance of NEP expression in early PC is a field of controversies between studies regarding differences in localization and level of expression patterns as well as differences in correlation or lack thereof with clinicopathologic parameters, including PSA relapse-free survival. Loss or decreased expression of NEP was observed in 219 prostatectomy specimens of patients with PC compared with normal prostate and prostatic intraepithelial neoplasia (PIN), although no correlation of NEP expression with clinical parameters (Gleason score and pathologic stage) or outcome (biochemical recurrence) was observed in these series [15]. In another series of 223 patients, loss of membranous NEP expression was significantly associated with a shorter time to PSA relapse [16]. In contrast, high NEP expression was associated with higher PSA recurrence in a cohort of 87 patients [17], in which 34 patients were lymph node positive and were not included in the recurrence analysis. The most extensive series investigating NEP as a predictor of PC reports on 2385 patients treated by RP in a single institution during a 14-year time period [18]. In these series, the authors observed a PSA recurrence-free survival decline from membranous over membrane-cytoplasmic to exclusively cytoplasmic NEP expression [18]. However, the best prognosis was observed with NEP negativity, which is a finding against the results of several preclinical models [6, 19], including ours [20] and is also at odds with the results of Osman et al. [16], with the rest of the results of this same series predicting a worse prognosis for a nonfunctional localization of NEP as well as the results of our present study. One could hypothesize that the mechanism promoting methylation of the NEP promoter and NEP expression loss inhibits also expression of cancer-promoting genes with the net effect being an improvement in prognosis. Alternatively, an artifact of loss of extracellular NEP during tissue processing in at least part of the samples remains a possibility. Importantly, staining intensity was not considered for analysis in their study.

Our findings regarding the clinical usefulness of NF*κ*B corroborate previous data indicative of association between NF*κ*B expression and grade [21, 22] and significant improvement of the prediction of outcome with the combination of Gleason score and nuclear NF*κ*B staining [23]. NF*κ*B has also been reported as an independent prognostic indicator of recurrence in groups with positive SMs [24], or lymph node invasion [25]. A few reports have emphasized on subcellular NF*κ*B compartmentalization, with reproducible results regarding the correlation of either cytoplasmic [26], or nuclear immunoreactivity [27] with PSA failure. In general, despite differences in localization between studies, high NF*κ*B is correlated with inferior outcome.

An issue that has been so far underestimated, despite its already established preclinical significance, concerns the potential interrelations between expression of NEP, ET-1, and NF*κ*B in early PC. In this study we coexamined all three parameters together at the clinical level and demonstrated that NEP expression is related to ET-1 and NF*κ*B expression, with the majority of high NEP-expressing cells demonstrating a low ET-1 and NF*κ*B expression level and reversely.

In multivariate analysis including preoperative PSA, tumor stage, grade, SMs, ET-1, and NF*κ*B expression, only pT stage, SMs, and NEP retained their importance in predicting PSA recurrence after RP. In fact, the risk of PSA relapse was approximately 4.386-fold greater in patients with low NEP expression compared to men with high expression of membranous NEP. In addition, this risk was greater than the risk derived from advanced pT stage (3.025 fold) and positive SM status (2.061 fold). These data confirm that there is a strong association between NEP expression and PSA relapse in hormone naïve PC patients and a strong rational for integration of NEP in the armamentarium of existing prognostic tools for early prediction of PC recurrence. Intriguingly, the emergence of significant NEP, ET-1, and NF*κ*B interrelations (Table 3) illustrates the clinical relevance of two pathways that might be in close communication. Our results implicate the existence of two clinicopathological patterns of hormone naïve PC with regard to the time of PSA recurrence after RP. The first pattern is featuring high NEP expression and low ET-1 and NF*κ*B expression and predicts a prolonged PSA relapse-free survival compared to the second one, which involves attenuation of NEP expression accompanied by elevated ET-1 and NF*κ*B levels and is characterized by a shortened PSA relapse-free survival.

Indeed, at the molecular level, there seems to be a functional link between the NEP/NPs and NF*κ*B pathways. NF*κ*B has been suggested as a mediator of the antiapoptotic actions of NPs, given that prosurvival signaling initiated by NPs involves activation of the kinases PI3K and Akt, which then activates NF*κ*B by phosphorylating its inhibitor, I*κ*B [28, 29]. In a recent *in vitro* work, we have revealed an inverse baseline expression pattern of the NEP/NPs and NF*κ*B/proteasome pathways in androgen-dependent and androgen-independent PC cells [20]. Further we have observed that NPs were able to induce NF*κ*B nuclear translocation and DNA binding, an event that was much more pronounced in androgen-independent cell lines that display low levels of NEP. These events were reversed by NP inhibitors (Patrikidou et al. unpublished data). The current study is the first linking, at a clinical level, NEP expression with ET-1 and NF*κ*B expression. These results complement our *in vitro* data on PC cell lines.

Nevertheless, several limitations of the present study should be considered. It should be acknowledged that the small number of patients included in the present study does not permit to draw unequivocal conclusions. Further, lowest Gleason scores (2, 3, and 4) are not represented in the study population and this might also blunt the validity of our results, although it might be hypothesized that differences between expression of NEP, ET-1, and NF*κ*B might be even more pronounced based on the underlying biology of the disease in its earliest phase. The evaluation of SMs, although clearly defined may be either misinterpreted due to the presence of crush, thermal, and electrocautery artifacts [30] or clinically less relevant in locally advanced disease [31]. Retrospective review of our prospectively collected data and the relatively short median followup of patients may have also biased our results. Moreover, the prognostic values of NEP, ET-1, and NF*κ*B might have been better established if they had been compared to predicted outcomes of validated nomograms. Finally, our study was not intended to be all inclusive of current prognostic markers such as seminal vesicle involvement, tumor marker ploidy status, and proliferation indexes.

 In conclusion, our study was designed in an effort to offer an integrated approach of the role of the NEP/endothelin and NF*κ*B pathways in the clinical course of PC patients. The relationship between recurrence (given the heterogeneity of the population), stage and grade of the tumors, and outcome was an exploratory (hypothesis generating) analysis and the primary goal of this study was to demonstrate a relationship between NEP/ET-1 and NF*κ*B signaling. Although further prospective evaluation to confirm this interaction is required, the strong preclinical model of PC evolution based on aberration of both NEP/NPs and NF*κ*B pathways seems to play a role in the clinical setting. Most importantly, the significant interrelations that we have suggested to exist between these two systems, if confirmed in a large prospective cohort, might encourage not only the incorporation of their pathological assessment into the current model of predictive factors but also the concept of their concurrent pharmacological inhibition to enable a greater therapeutic benefit in tumors with aggressive biology.

## Figures and Tables

**Table 1:** Patients' clinicopathological characteristics (*n* = 70) and associations with time to PSA relapse.

Characteristic	Subgroup	*n* (%)	*P*
Age (years)	≤65	31 (44.3)	0.277
range 47–75	>65	39 (55.7)	
pre-op PSA (ng/mL)	<10	60 (85.7)	0.143
range 2.8–23.9	≥10	10 (14.3)	
pT stage	≤2	42 (60.0)	<0.001
	≥3	28 (40.0)	
Gleason score	≤7 (3 + 4)	50 (46.8)	<0.001
	≥7 (4 + 3)	20 (44.2)	
SMs	(−)	49 (70.0)	0.004
	(+)	21 (30.0)	
LN status	N0	53 (75.7)	0.072
	N1	6 (8.6)	
	N*x *	11 (15.7)	

Pre-op PSA: preoperative PSA; pT stage: pathologic TNM stage; SMs: surgical margins; LN: lymph node; *n*: number of patients; *P*: statistical value.

**Table 2:** Correlations between levels of NEP, ET-1, NF*κ*B expression, and clinicopathological characteristics.

Characteristic	NEP		*P*	ET-1		*P*	NF*κ*B		*P*
	low	high		low	high		low	high	
	*n (%)*	*n (%)*		*n (%)*	*n (%)*		*n (%)*	*n (%)*	
Pre-op PSA									
<10	28 (96.6)	34 (82.9)	0.076	32 (94.1)	30 (83.3)	0.277	35 (83.3)	27 (96.4)	0.191
≥10	1 (3.4)	7 (17.1)		2 (5.9)	6 (16.7)		7 (16.7)	1 (3.6)	
Gleason score									
≤7 (3 + 4)	20 (55.6)	30 (88.2)	0.003	19 (55.9)	31 (86.1)	0.008	37 (74)	6 (30)	0.001
≥7 (4 + 3)	16 (44.4)	4 (11.8)		15 (44.1)	5 (13.9)		13 (26)	14 (70)	
pT stage									
≤2	17 (47.2)	25 (73.5)	0.030	14 (41.2)	28 (77.8)	0.003	32 (76.2)	11 (39.3)	0.003
≥3	19 (52.8)	9 (26.5)		20 (58.8)	8 (22.2)		10 (23.8)	17 (60.7)	
SMs									
(−)	22 (61.1)	27 (79.4)	0.121	20 (58.8)	29 (80.6)	0.068	33 (76.7)	16 (59.3)	0.180
(+)	14 (38.9)	7 (20.6)		14 (41.2)	7 (19.4)		10 (23.3)	11 (40.7)	

Pre-op PSA: preoperative PSA; pT stage: pathologic TNM stage; SMs: surgical margins; *n*: number of patients; *P*: statistical value.

**Table 3:** Correlations between NEP, ET-1, and NF*κ*B expression.

Variables	NEP		HR [95% CI]	*P*
	Low	High		
	*n (%)*	*n* (%)		
ET-1				
Low	3	31	0.016 [0.003–0.071]	<0.001
High	31	5		
NF*κ*B				
Low	10	33	0.012 [0.001–0.097]	<0.001
High	26	1		

*n*: number of patients; HR: hazard ratio; CI: confidence interval; *P*: statistical value.

**Table 4:** Multivariate Cox regression analysis.

Variable	Groups	HR [95% CI]	*P*
NEP	low vs high	4.386 [1.305–14.706]	0.017
ET-1	low vs high	0.636 [0.167–2.422]	0.507
NF*κ*B	low vs high	0.851 [0.273–2.652]	0.781
pre-op PSA	<10 vs ≥10	0.334 [0.101–1.097]	0.071
Gleason score	≤7 (3 + 4) vs ≥7 (4 + 3)	0.637 [0.276–1.473]	0.291
pT stage	≤2 vs ≥3	0.330 [0.137–0.795]	0.013
SMs	(−) vs (+)	0.485 [0.247–0.953]	0.036

pT stage: pathologic TNM stage; SMs: surgical margins; pre-op PSA: preoperative PSA; HR: hazard ratio; CI: confidence interval; *P*: statistical value; vs = versus.
